# Intermolecular masking of the HIV-1 Rev NLS by the cellular protein HIC: Novel insights into the regulation of Rev nuclear import

**DOI:** 10.1186/1742-4690-8-17

**Published:** 2011-03-14

**Authors:** Lili Gu, Takahiro Tsuji, Mohamed Ali Jarboui, Geok P Yeo, Noreen Sheehy, William W Hall, Virginie W Gautier

**Affiliations:** 1 UCD-Centre for Research in Infectious Diseases, School of Medicine and Medical Science, University College Dublin (UCD), Belfield, Dublin 4, Ireland; 2 Host-Pathogen Interaction Lab, Institute of Immunology/Department of Biology, National University of Ireland Maynooth, Maynooth, Co. Kildare, Ireland; 3 Department of Pathology, National Institute of Infectious Diseases, Gakuen 4-7-1, Musashimurayama, Tokyo, Japan

## Abstract

**Background:**

The HIV-1 regulatory protein Rev, which is essential for viral replication, mediates the nuclear export of unspliced viral transcripts. Rev nuclear function requires active nucleocytoplasmic shuttling, and Rev nuclear import is mediated by the recognition of its Nuclear Localisation Signal (NLS) by multiple import factors, which include transportin and importin β. However, it remains unclear which nuclear import pathway(s) predominate *in vivo*, and the cellular environment that modulates Rev nucleocytoplasmic shuttling remains to be characterised.

**Results:**

In our study, we have identified the cellular protein HIC (Human I-mfa domain-Containing protein) as a novel interactor of HIV-1 Rev. We demonstrate that HIC selectively interferes with Rev NLS interaction with importin β and impedes its nuclear import and function, but does not affect Rev nuclear import mediated by transportin. Hence, the molecular determinants mediating Rev-NLS recognition by importin β and transportin appear to be distinct. Furthermore, we have employed HIC and M9 M, a peptide specifically designed to inhibit the transportin-mediated nuclear import pathway, to characterise Rev nuclear import pathways within different cellular environments. Remarkably, we could show that in 293T, HeLa, COS7, Jurkat, U937, THP-1 and CEM cells, Rev nuclear import is cell type specific and alternatively mediated by transportin or importin β, in a mutually exclusive fashion.

**Conclusions:**

Rev cytoplasmic sequestration by HIC may represent a novel mechanism for the control of Rev function. These studies highlight that the multivalent nature of the Rev NLS for different import receptors enables Rev to adapt its nuclear trafficking strategy.

## Background

The HIV-1 regulatory protein Rev (18 kDa) is essential for HIV-1 replication [1,2]. Rev is predominantly localised in the nucleus/nucleolus [3], and its primary function is to mediate the nuclear export of partially spliced and unspliced viral transcripts. Rev has also been shown to modulate splicing and translation of viral transcripts, and their subsequent packaging, and to interfere with integration of the HIV-1 genome [4-7]. Rev nuclear export of unspliced viral transcripts requires active shuttling of the protein between the nucleus and cytoplasm via nuclear pore complexes (NPCs) which is mediated by two major functional domains, the Nuclear Localisation Signal (NLS) and the Nuclear Export Signal (NES) [8,9]. The leucine-rich Rev NES binds directly to CRM1, which in concert with DDX3, a DEAD box RNA helicase, facilitates Rev nuclear export of unspliced viral transcripts via the NPC [10-14]. Also, Rev-export function was shown to be inhibited by Nuclear Factor 90 (NF90)[15]. The basic arginine-rich Rev NLS mediates both Rev nuclear import and binding to the Rev Response Element (RRE), a *cis*-acting RNA element present in all unspliced viral transcripts [16-18]. The Rev NLS is recognized by at least 5 different importin β family members, including importin β, transportin, importin 5, importin 7 and importin 9, which facilitate its nuclear import [19-23]. Despite evidence showing the utilisation of multiple nuclear import receptors *in vitro *by Rev, it remains unclear if some are redundant and/or if, under specific conditions, one nuclear import pathway may predominate *in vivo*. Hutten *et al. *described transportin as a major Rev nuclear import receptor [23]. However, this study was restricted to HeLa cells, and the molecular and cellular determinants governing the interaction of Rev with one or the other nuclear import receptors have not been investigated.

In this report, we identified a novel interaction between HIV-1 Rev and the cellular protein HIC (Human I-mfa domain-Containing protein). HIC is a 246 amino acid protein with a prominently cytoplasmic distribution. The cysteine-rich C-terminal domains of HIC and the Inhibitor of MyoD family a (I-mfa) share a high homology (74%) and are essential for their activities [24]. HIC acts as a regulator of transcription and interacts with and/or modulates the activity of several cellular and viral transcription factors, including Axin, cyclin T1 and T2, TCF1, HIV-1 Tat, HTLV-1 Tax and KSHV LANA [24-29]. We previously reported that the ectopic expression of HIC resulted in the mislocalisation of HIV-1 Tat to the cytoplasm. This contrasted with an earlier report which showed that Tat and HIC co-localised in the nucleolus [28]. Nevertheless, these studies were descriptive, and the effects of HIC on the nuclear transport machinery were not investigated.

In this report, we explored the mechanisms whereby HIC could regulate Rev nuclear import and demonstrated that HIC selectively blocks importin β- but not transportin-mediated Rev nuclear import via a mechanism involving the intermolecular masking of Rev NLS by HIC. In addition, we employed HIC, as an inhibitor of importin β mediated Rev nuclear import, and M9 M, a peptide which specifically inhibits the transportin pathway, as tools to further characterise Rev nuclear import pathway(s) in HeLa, 293T, COS7, Jurkat, CEM, THP-1 and U937 cells. While we confirmed that transportin is the major import receptor for Rev in HeLa, THP-1 and U937 cells, we showed that in 293T, COS7, CEM and Jurkat cells, importin β-mediated Rev nuclear import is dominant. Subsequently, reporter gene assays revealed that HIC contributes to the control of Rev function in 293T Jurkat and CEM but not in HeLa, U937 and THP-1 cells. Collectively these results demonstrate that Rev nuclear import is tightly regulated and suggest that the molecular determinants mediating Rev transport by importin β and transportin are distinct, and that the Rev dominant nuclear import pathway is cell type specific.

## Results

### HIC sequesters Rev in the cytoplasm by inhibiting its nuclear import *in vivo*

**Figure 1:**
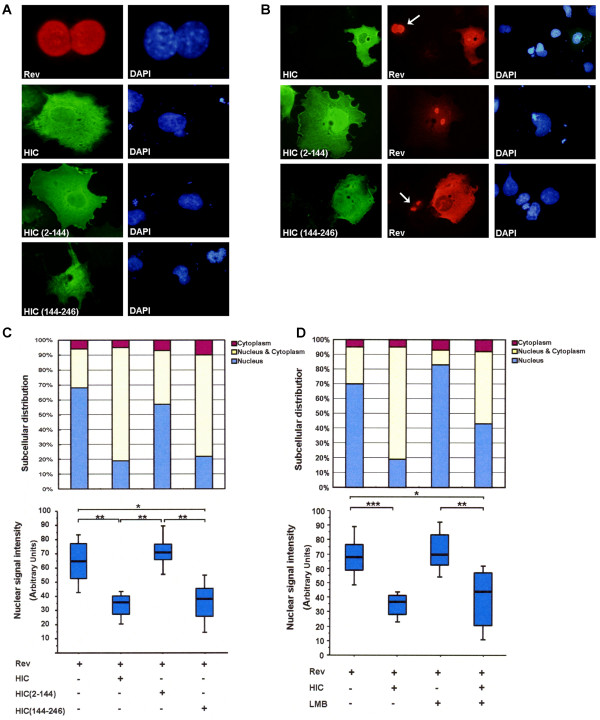
**HIC sequesters HIV-1 Rev in the cytoplasm by inhibiting its nuclear import *in vivo***. COS7 cells were transiently transfected with HA-Rev; pFLAG-HIC; pFLAG-HIC (2-144); pFLAG-HIC (144-246). Rev expression is shown in Red and HIC, HIC (2-144) and HIC (144-246) expression is shown in Green. Nuclei were counterstained with DAPI (Blue). Representative images of transfected cells are shown. Arrows indicate cells expressing Rev only. **(A) Localisation of Rev, HIC and its mutants in singly transfected COS7 cells. (B) Co-expression of HIV-1 Rev and HIC or HIC (144-246) results in the redistribution of Rev to the cytoplasm. (C) Quantitative analysis of Rev nuclear localization**. Same conditions as described in B. Upper panel: quantitative analysis of Rev subcellular localisation. A minimum of 100 transfected cells was counted per well and results are expressed as a percentage of the total number of cells counted according to the classification: nucleus-dominant (blue), nucleus/cytoplasm-equivalent (yellow), or cytoplasm-dominant (red). Lower panel: quantitative analysis of Rev nuclear signal. Rev nuclear signal intensities were analyzed by Image J (NIH) from a minimum of 100 transfected cells and shown by box plots. Statistical significance analysis was performed with a two-tailed unpaired Student's *t *test *, *P *< 0.05; **, *P *< 0.01. **(D) HIC retains Rev in the cytoplasm by inhibiting its nuclear import**. COS7 cells were transfected with HA-Rev, and/or pFLAG-HIC and incubated with or without 20 nM Leptomycin B (LMB) for 3 hours. Upper panel: quantitative analysis of Rev subcellular localisation. Lower panel: quantitative analysis of Rev nuclear signal.

We performed colocalisation studies in COS7 cells transfected with HIC, its mutants and Rev. The localization of HIC and the mutant, HIC (2-144) was primarily cytoplasmic, although they could be detected in the nucleus (Figure 1A). Interestingly, HIC (144-246) was localized widely in the cytoplasm and nucleus in a diffuse manner (Figure 1A). The majority of cells expressing Rev did so exclusively in the nucleus and/or nucleolus (68%), while in the remainder, Rev was present in the cytoplasm only, or diffusely in the cytoplasm and nucleus (Figure 1A, B, and 1C, upper column). In contrast, when Rev was co-expressed with HIC or HIC (144-246) containing the I-mfa domain, both colocalised in the cytoplasm and the percentage of cells displaying Rev in the nucleus was significantly reduced (19% and 22% for HIC and HIC (144-246), respectively) (Figure 1A, B, and 1C, upper panel). Interestingly, HIC (2-144) did not co-localise with Rev, or influence its nuclear localisation (57%) (Figure 1A, B, and 1C, upper panel). To further evaluate if the cytoplasmic redistribution of Rev by HIC is associated with a reduction in Rev nuclear accumulation, we quantified Rev nuclear signal intensity with the ImageJ 1.41 software (NIH) on 100 cells expressing Rev in the presence or absence of HIC and its mutants. We could observe a significant reduction (48%) in Rev nuclear signal intensity when Rev was co-expressed with HIC or HIC (144-246), while HIC (2-144) did not influence Rev nuclear signal intensity (Figure 1D, lower panel). Therefore, the ectopic expression of HIC results in the cytoplasmic redistribution of Rev with a concomitant reduction in its nuclear accumulation and this effect was dependent on the I-mfa domain.

To determine whether HIC inhibits Rev nuclear import or promotes Rev nuclear export, we repeated the colocalisation studies, in the presence of Leptomycin B, which specifically inhibits nuclear export mediated by CRM1 [11]. Since Rev localisation is the result of a net balance between Rev import and export, treatment with LMB interrupted the nuclear export and resulted in an overall increase in Rev nuclear localisation, as determined by an increase in both the number of cells displaying Rev exclusively in the nucleus and Rev nuclear signal intensity (Figure 1D). However, LMB did not prevent the overall effects of HIC on Rev cytoplasmic redistribution. Indeed when Rev was co-expressed with HIC, the percentage of cells displaying Rev in the nucleus decreased from 83% (Rev alone+LMB) to 43% (Rev+HIC+LMB) (Figure 1D upper panel). In addition, the Rev nuclear signal intensity also decreased from 72% (Rev alone+LMB) to 40% (Rev+HIC+LMB) (Figure 1D, lower panel). These observations support the view that HIC most likely sequesters Rev in the cytoplasm by inhibiting its nuclear import rather than promoting its nuclear export.

### HIV-1 Rev nuclear import mediated by importin β is selectively blocked *in vitro *by a competitive excess of HIC

**Figure 2:**
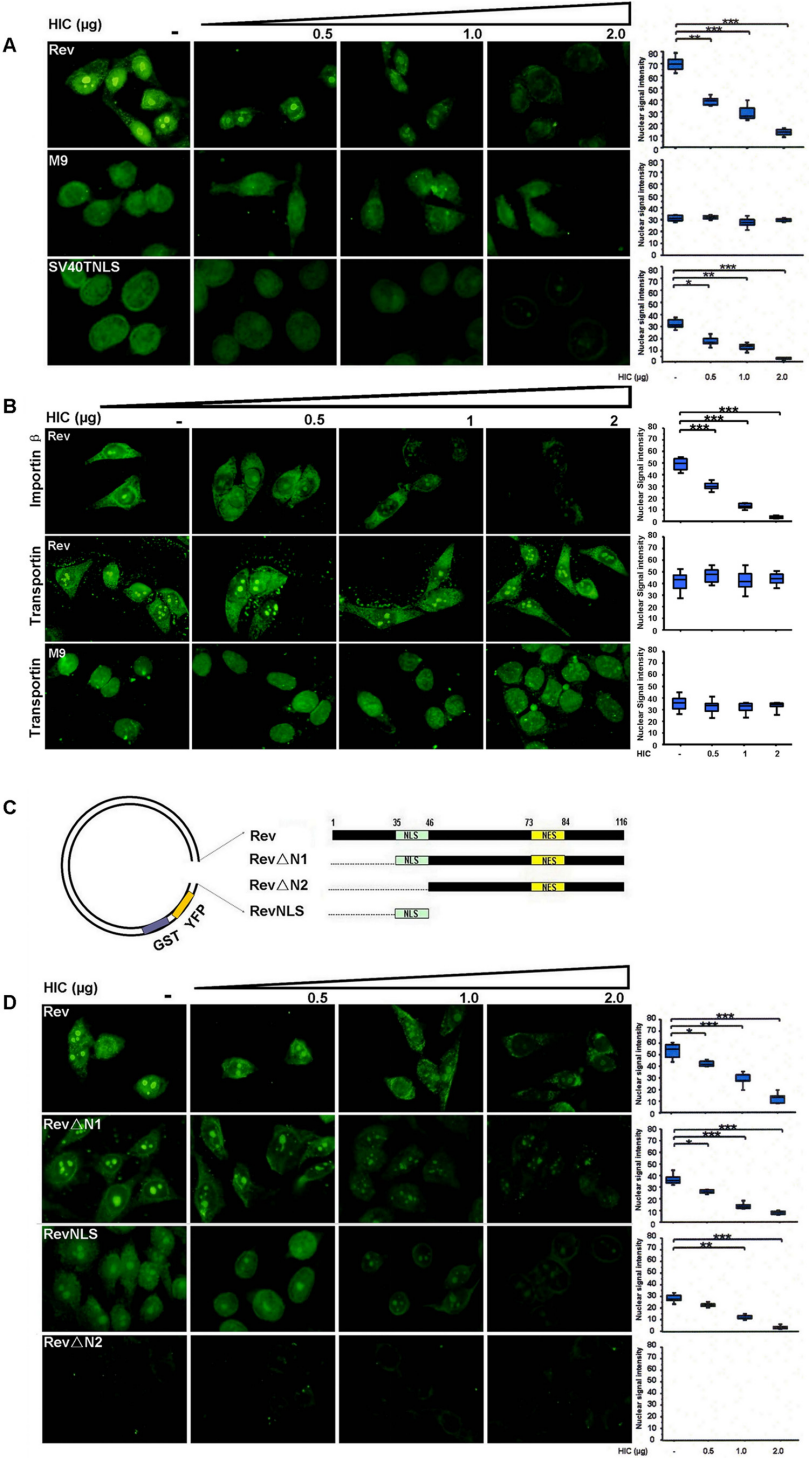
**HIV-1 Rev nuclear import mediated by importin β is selectively blocked *in vitro *by competitive excess of HIC**. Nuclear import of GST-YFP-Rev, GST-SV40TNLS-GFP and GST-YFP-M9 was examined using *in vitro *nuclear import assays. Digitonin permeabilised HeLa cells were incubated with 10 μl of reaction mixtures containing 1 μg of import substrate, ATP regeneration system and nuclear import factors. Recombinant 6×His-HIC (0.5, 1 or 2 μg) was added to investigate the effect on Rev nuclear import. In all the cases, Rev nuclear signal intensities were analyzed by ImageJ for a minimum of 100 cells and illustrated by box plots (arbitrary units). Statistical significance analysis was performed with a two-tailed unpaired Student's *t *test *, *P *< 0.05; **, *P *< 0.01; ***, *P *< 0.001 **(A) Recombinant HIC protein abolishes Rev nuclear import**. Rabbit Reticulocyte Lysate (RRL) was employed as source of multiple import factors. **(B) HIC specifically inhibits Rev nuclear import mediated by importin β but not by transportin**. 2 μg of recombinant importin β or transportin was employed as the only source of import factor. **(C) Schematic representation of HIV-1 Rev and deletion mutants. (D) Rev NLS domain is necessary and sufficient for Rev nuclear import inhibition by HIC**. Nuclear imports of GST-YFP-Rev, GST-YFP-RevΔN1, GST-YFP-RevΔN2 and GST-YFP-RevNLS were examined using *in vitro *nuclear import assays. Importin β was employed as the only source of nuclear import factor.

To examine the mechanisms regulating Rev nuclear import, we performed *in vitro *nuclear import assays, where a competitive excess of the cellular recombinant protein HIC was employed. HeLa cells were treated with digitonin, which selectively permeabilises the cytoplasmic membrane and rabbit reticulocyte lysate (RRL) was employed as a source of import factors with recombinant GST-YFP-Rev, GST-YFP-M9 or GST SV40TNLS-GFP being used as fluorescent import substrates. M9, which is imported into the nucleus by transportin, independently of the importin α/β pathway, and SV40T NLS, which is imported to the nucleus by the importin α/β pathway, were employed as controls [30,31]. We first confirmed that the nuclear import of the substrates was actively and selectively mediated by cellular factors through the NPC, in an energy and RanGTP-dependent manner (data not shown). Subsequently, addition of competitive and increasing amounts of HIC recombinant protein (0.5-2 μg) resulted in a decreased signal intensity in the nucleus for Rev and for SV40T NLS, in a dose dependent manner (Figure 2A). In contrast, HIC did not affect M9 nuclear import (Figure 2A), demonstrating that Rev and SV40T NLS nuclear import is selectively and efficiently inhibited by HIC and is not the result of a general block of nuclear import pathways or obstruction of the NPC.

To further dissect the mechanisms underlying HIC inhibition of Rev nuclear import, we reduced the complexity of this system, by employing 2 μg of recombinant importin β or transportin, as sole import factors. Remarkably, HIC efficiently and distinctively abolished Rev nuclear import mediated by importin β but not by transportin (Figure 2B). As previously shown, HIC did not block transportin mediated nuclear import of M9. To further examine the effect of HIC on importin β-mediated Rev import, we performed similar experiments with Rev mutants (GST-YFP-RevΔN1/-RevΔN2/-RevNLS) with or without deletions of the Rev NLS domain (Figure 2C). Strong fluorescent signals were observed in the nucleus for both RevΔN1 and RevNLS, both of which encompass the functional NLS sequence, while RevΔN2 lost its nuclear localisation due to the absence of the NLS domain (Figure 2C). The addition of increasing amounts of HIC (0.5-2 μg) correlated with a decreasing nuclear signal intensity for both RevΔN1 and RevNLS (Figure 2C), demonstrating that the RevNLS is sufficient and necessary for both Rev nuclear import and its inhibition by HIC.

### HIV-1 Rev and HIC interact directly *in vitro *and form a complex *in vivo*

**Figure 3:**
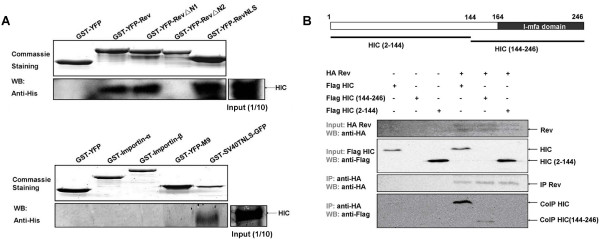
**HIV-1 Rev and HIC interact directly *in vitro *and form a complex *in vivo***. **(A) GST pull-down assays show that Rev NLS (upper panel) and SV40T NLS (lower panel) interact directly with HIC *in vitro*. **Purified recombinant HIC protein was incubated with immobilised GST-YFP (control) and various GST-YFP fusions proteins (bait). Interacting proteins were subsequently eluted and resolved by SDS-PAGE. HIC was detected by Western Blot analysis and Commassie staining indicated the quantity and quality of GST fusion proteins employed. **(B) HIV-1 Rev and HIC form a complex *in vivo***. 293T cells were transiently transfected with HA-Rev, pFLAG-HIC/-HIC (2-144)/-HIC (144-246). Input and immunoprecipitates were analysed by Western-Blot (WB) to examine expression levels of Rev, HIC and its mutants, and co-immunoprecipitation of HIC, HIC (144-246) and HA-Rev, respectively. Similar to previous studies and for reasons that remain unclear HIC mutant (144-246) expression was difficult to detect in the input [25,28].

We examined whether HIC-mediated inhibition of Rev nuclear import involves a direct interaction by performing *in vitro *GST-pull downs with recombinant HIC and GST-YFP (control), or GST-YFP fusion proteins (GST-YFP-Rev/-RevΔN1/-RevΔN2/-RevNLS). HIC was specifically detected in the eluted fractions of GST-YFP Rev/-RevΔN1/-RevNLS, all of which contained the NLS domain, whereas there was no association between HIC and GST-YFP or GST-YFP-RevΔN2, which lack the NLS domain (Figure 3A). These results demonstrate that HIC physically interacts with Rev and that the Rev NLS is sufficient and necessary to mediate this interaction. Similarly, we conducted *in vitro *GST-pull down assays, which demonstrated that SV40T NLS, but not importin α, importin β or M9 directly interacts with HIC (Figure 3A) and this again excluded the possibility that HIC mediates a general nuclear import block by physically targeting the import factors.

Subsequently, co-immunoprecipitation assays were employed to investigate potential interactions between HIV-1 Rev and HIC in transfected 293T cells. HA-Rev was immunoprecipitated and HIC was specifically detected in the eluted fraction (Figure 3B). Similarly, the HIC mutant (144-246), which contains the I-mfa domain, but not the HIC mutant (2-144), interacted with Rev (Figure 3B). Thus, HIC and Rev interact *in vivo *and *in vitro*, and this interaction is mediated by the I-mfa domain of HIC and Rev NLS domain.

### HIC selectively interferes with the Rev NLS interaction with importin β

**Figure 4:**
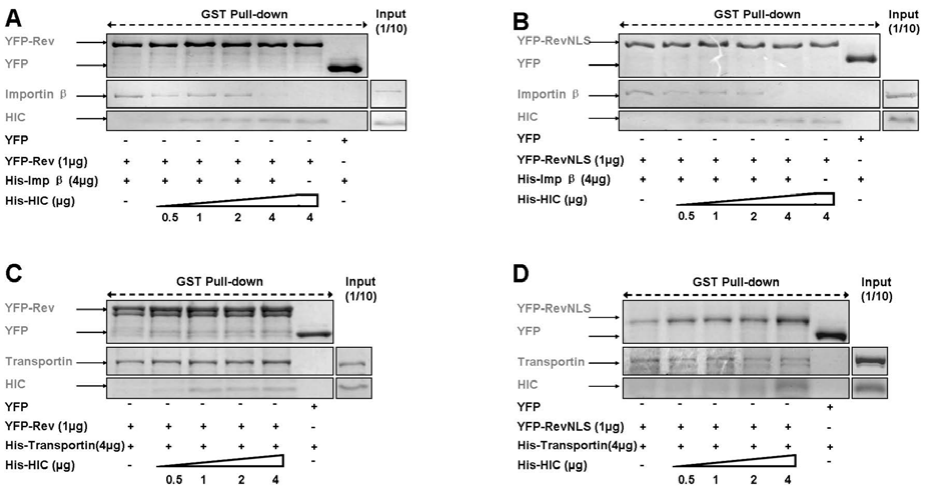
**HIC interferes with HIV-1 Rev molecular recognition by importin β *in vitro***. Immobilised GST-YFP-Rev/-Rev NLS were incubated with 6xHis-importin β or 6xHis-transportin and 0.5-4 μg of 6xHis-HIC. Following GST pull-down assays, Commassie staining shows that HIC specifically competes with the binding of Rev (A) or Rev NLS (B) to importin β. HIC does not interfere with the binding of Rev (C) or Rev NLS (D) to transportin.

We next assessed whether competition between HIC and importin β for Rev binding might account for the observed reduced nuclear import and performed *in vitro *binding assay in which HIC with either importin β or transportin compete for GST-YFP-Rev binding (Figure 4A, B). HIC selectively interfered with the binding of Rev to importin β but not transportin in a dose dependent manner. Indeed, maximum amounts of HIC recombinant protein were sufficient to fully abolish the binding of importin β to Rev. Remarkably, similar results were obtained when using Rev-NLS (Figure 4C, D). Of note, Rev could bind simultaneously to both HIC and transportin.

### The dominant Rev nuclear import pathway is cell type dependent

**Figure 5:**
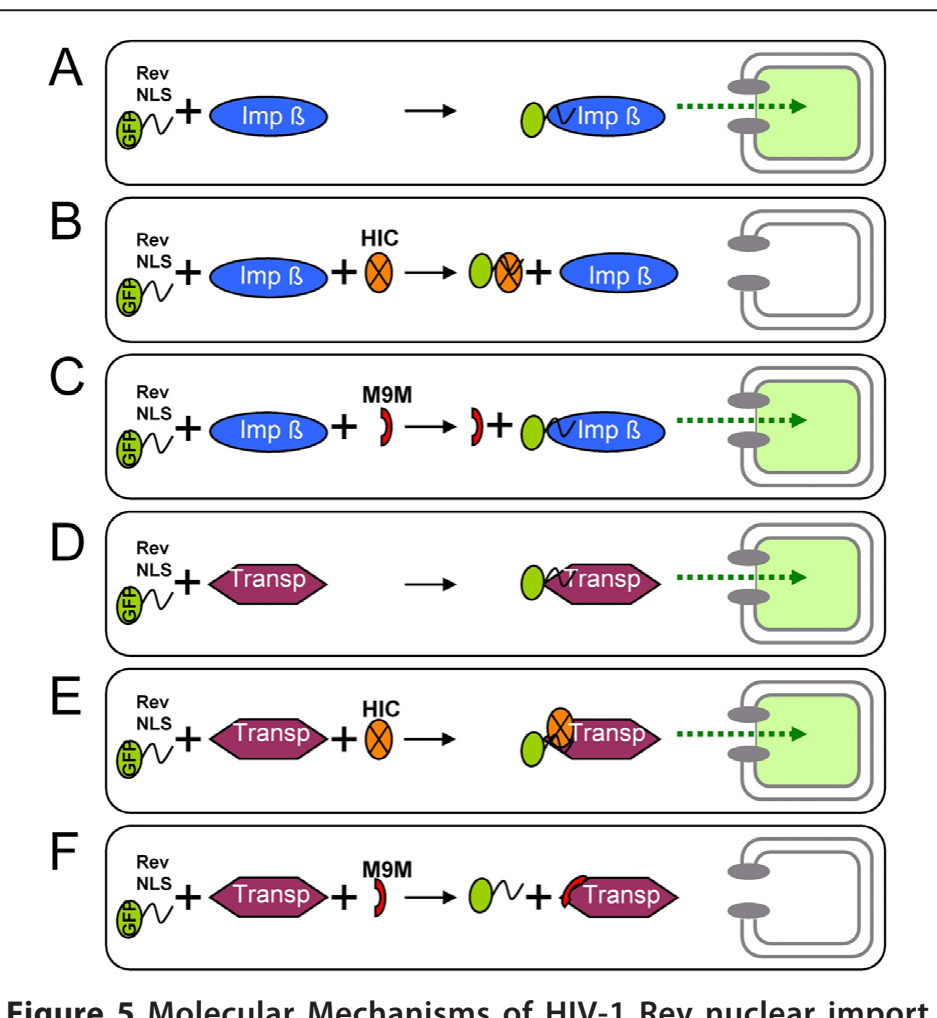
**Molecular Mechanisms of HIV-1 Rev nuclear import inhibition by HIC and M9M**. (A) Rev nuclear import mediated by importin β. (B) HIC interferes with the interaction of Rev NLS with importin β and as a results impedes Rev nuclear import. (C) M9 M does not interfere with importin β-mediated Rev nuclear import. (D) Rev nuclear import mediated by transportin. (E) Rev binds simultaneously to both HIC and transportin, which mediate its nuclear import. (F) M9 M tightly interacts with transportin and inhibits transportin mediated Rev nuclear import.

**Figure 6:**
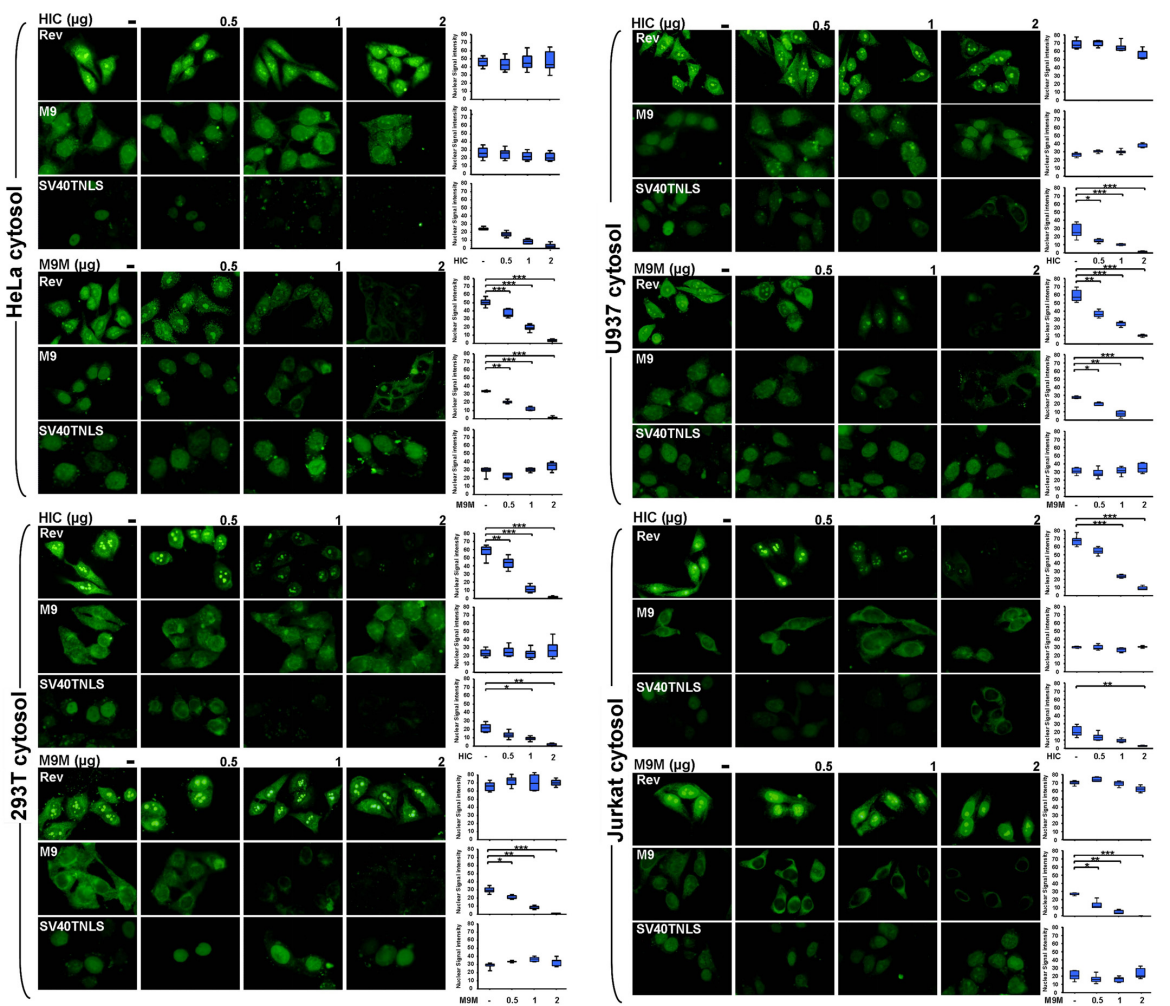
**HIV-1 Rev dominant nuclear import pathways are cell specific**. Nuclear import of GST-YFP-Rev, GST-YFP-M9 and GST-SV40T NLS-GFP were examined using *in vitro *nuclear import assays. Digitonin permeabilized HeLa cells were incubated with 10 μl of reaction mixtures containing 1 μg of an import substrate, ATP regeneration system, and 293T/HeLa/U937/Jurkat cytosolic extracts. Recombinant 6×His-HIC at 0.5, 1 or 2 μg was added. Rev nuclear signal intensities were analyzed by ImageJ for a minimum of 100 cells and illustrated by box plots. Statistical significance analysis was performed with a two-tailed unpaired Student's *t *test *, *P *< 0.05; **, *P *< 0.01; ***, *P *< 0.001

Hutten *et al. *have reported that transportin, but not importin β, could mediate Rev nuclear import in HeLa cells [23]. Here, we use selective inhibition of importin β-mediated Rev nuclear import by HIC and the M9 M peptide, the latter which specifically inhibits the transportin pathway to further characterise Rev dominant nuclear import pathway(s) [32] (Figure 5). We employed HeLa, 293T, COS7, U937 (monocytic leukemia), Jurkat (E6-1 clone; T lymphocyte), THP-1 (Acute monocytic leukemia) and CEM (T cell leukemia) cell cytosolic extracts as seven distinct sources of import receptors in our *in vitro *nuclear import assays (Figure 6 and Figure S1; Additional File 1). First, we established that (i) both the importin β and transportin pathways were functional in the seven cell lines tested, as shown by the effective nuclear import of SV40T NLS and M9, and that (ii) all the cell types dysplayed relatively similar expression levels for transportin and importin β as revealed by Western Blot analysis of the different cytosolic fractions (Figure 6, Figure S1; Additional File 1 and Figure S2; Additional File 2). In parallel, endogenous HIC expression was observed in all the cell types except Jurkat cells (Figure S2; Additional File 2). Next, we confirmed in all the cell lines analysed, that HIC and M9 M could efficiently and selectively block the importin and transportin pathways respectively. Indeed, HIC inhibited SV40T NLS, but not M9 nuclear import, while M9 M inhibited M9, but not SV40T NLS nuclear import (Figure 6 and Figure S1; Additional File 1).

Remarkably, HIC selectively blocked Rev nuclear import in the presence of 293T, COS7, CEM or Jurkat cytosolic extracts (Figure 6 and Figure S1; Additional File 1). However, its nuclear import remained unaffected by HIC when HeLa, THP-1 or U937 cytosolic extracts were employed (Figure 6 and Figure S1; Additional File 1). In contrast, M9 M inhibited Rev nuclear import when HeLa, THP-1 or U937 cytosolic extracts were employed but did not affect Rev nuclear import mediated by 293T, COS7, CEM or Jurkat cytosolic extracts (Figure 6 and Figure S1; Additional File 1). These results are consistent with Hutten *et al. *[23] and support their finding that transportin is the major nuclear import receptor for Rev in HeLa cells. Additionally, our results demonstrated that transportin is also the primary import factor in U937 and THP-1 cells. In 293T, COS7, CEM and Jurkat however, the Rev dominant nuclear import pathway appears to involve importin β, which is consistent with our co-localisation assay performed in COS7 cells.

### HIC inhibits Rev function in a cell-specific fashion

**Figure 7:**
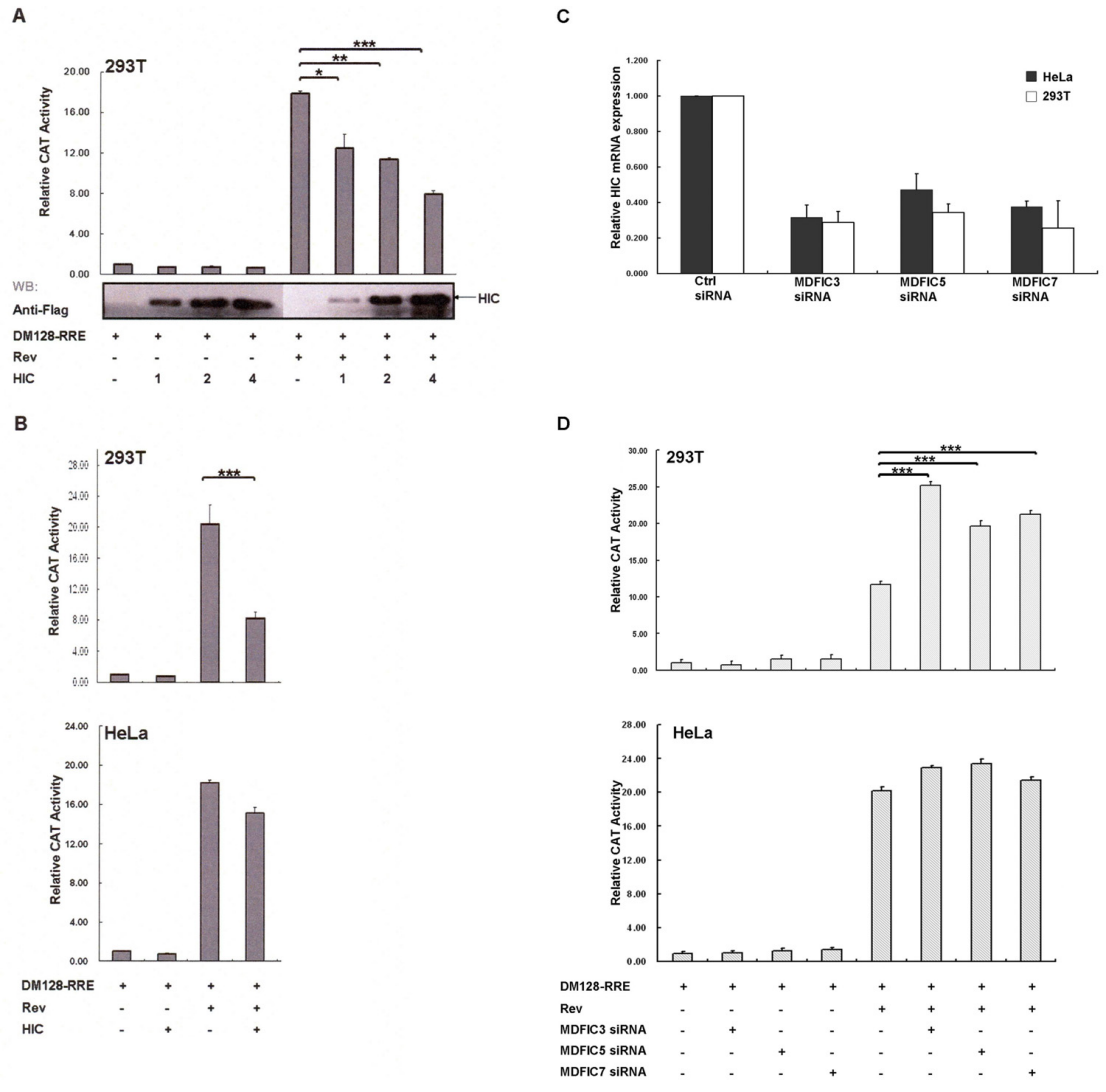
**HIC inhibits HIV-1 Rev function in a cell-specific fashion**. **A. HIC down-regulates Rev activity in a dose dependent manner**. 293T cells were transfected with 0.1 μg of pDM128-RRE combined with 0.05 μg of HA-Rev and 1, 2 or 4 μg of FLAG-HIC. Relative CAT activity is compared with 100% for Rev activity of pDM128-RRE. Values are mean ± standard deviation. Data are representative of a minimum of three independent experiments performed in duplicate. Statistical significance analysis was performed with a two-tailed unpaired Student's *t *test *, *P *< 0.05; **, *P *< 0.01; ***, *P *< 0.001**. B. The down-regulation of Rev activity by HIC is dependent on the I-mfa domain and is cell-specific**. 293T or HeLa cells were transfected with 0.1 μg of pDM128-RRE combined with 0.05 μg of HA-Rev and 4 μg of FLAG HIC. Relative CAT activity was analysed as described above. **C. Evaluation of siRNA knockdown of HIC**. Real-time RT-PCR analysis of HIC mRNA levels for HeLa and 293T cells was performed at 72 hours following reverse transfection of three distinct HIC siRNAs or with siRNAs directed against luciferase (GL2) as negative control. These experiments were each performed in duplicates and the mean average results are shown. **D. Effects of siRNA knockdown of HIC on Rev activity are cell-specific**. 293T or HeLa cells were first reverse-transfected with three independent HIC siRNAs (30pmoles) or negative control siRNA (30pmoles) and after 24 hours were transfected with 0.2 μg of pDM128-RRE, 0.02 μg RL-TK and 0.02 μg of HA-Rev or parent plasmid. Relative CAT activity was analysed as described above. Values are mean ± standard deviation. Data are representative of a minimum of three independent experiments performed in duplicate.

**Figure 8:**
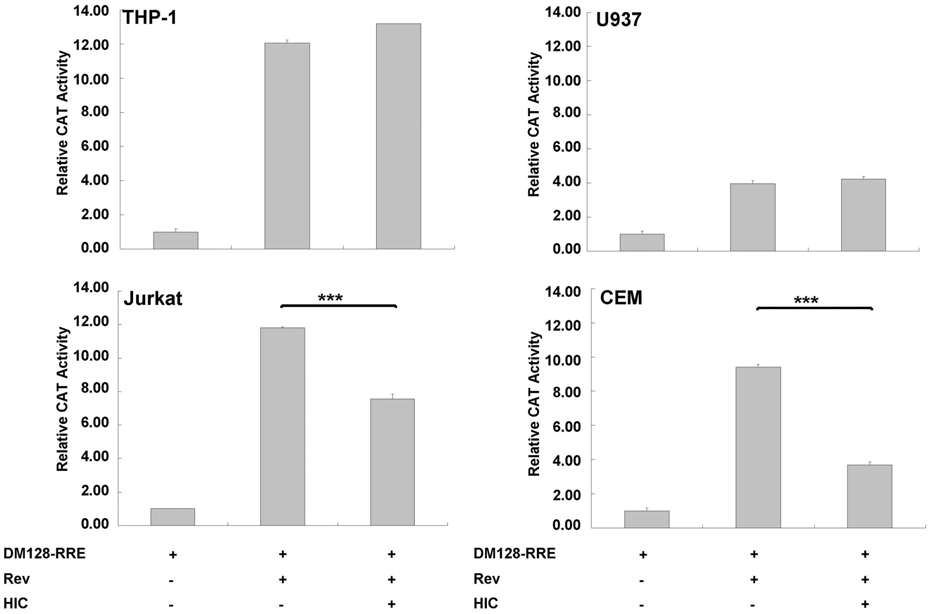
**HIC inhibits HIV-1 Rev function in Jurkat and CEM but not in U937 or THP-1**. Jurkat, CEM, U937 and THP-1 cells were transfected with 1 μg of pDM128-RRE combined with 0.5 μg of HA-Rev and 4 μg of FLAG HIC or parent plasmid. Relative CAT activity was analysed 24 hours post-transfection as described before. Values are mean ± standard deviation. Data are representative of a minimum of three independent experiments performed in triplicate.

We next sought to examine the biological relevance of HIC and Rev interaction on Rev activity employing the CAT reporter gene pDM128-RRE (28). The vector pDM128-RRE expresses transcripts consisting of a splicing donor site and a splicing acceptor site flanking the CAT gene and HIV-1 RRE *cis*-acting element. The unspliced transcripts are only exported to the cytoplasm in the presence of Rev, which ultimately results in the expression of the CAT protein (28). First, 293T cells were transfected with HA-Rev, FLAG-HIC and pDM128-RRE and assayed for CAT expression levels by ELISA. Rev induced a 17-fold increase in the CAT expression in 293T cells. HIC did not affect the CAT basal expression level (Figure 7A). In contrast, increasing levels of HIC expression were correlated with decreasing Rev activity in a dose-dependent manner. In 293T cells, maximal down-regulation of Rev activity corresponded to over 50% inhibition (Figure 7A). We then repeated the Rev functional assays with HIC in HeLa cells. Remarkably and in contrast to 293T cells, HIC had a modest effect (12% reduction) on Rev function (Figure 7B). Importantly, HIC overexpression did not modulate Rev expression levels, as monitored by WB analysis (Figure S3A; Additional File 3). We extended this functional assay to additional cell lines. Similarly, HIC down-regulated Rev function in Jurkat and CEM cells while in U937 and THP-1 cells, Rev activity remained unaffected by HIC overexpression (Figure 8). These results strongly suggest that HIC inhibits Rev activity in a cell-specific manner. Subsequently, to investigate the role of endogenous level of HIC expression on Rev activity, we performed small interfering RNA (siRNA)-mediated knockdown of HIC. As shown by quantitative real-time RT-PCR, all three independent HIC siRNAs, MDFIC_3, MDFIC_5 and MDFIC_7, effectively down-regulated HIC mRNA expression (Figure 7C), in HeLa and 293T cells with MDFIC_3 being the most effective siRNA (75% knockdown). These effects were also observed at the protein levels (Figure S3B; Additional File 3). In 293T, HIC knockdown resulted in a marked increase, up to 240%, in Rev activity but did not significantly affect Rev function in HeLa cells, demonstrating that an endogenous level of HIC expression interferes with Rev function in a cell type specific manner (Figure 7D). Importantly, HIC knock-down did not affect Rev expression levels as determined by WB analysis (Figure S3B; Additional File 3). Furthermore, the consistency of the observed effects, mediated by three independent siRNAs, strongly supports the view that these effects were specific and cannot be attributed to off-target silencing.

Collectively, these functional results correlate with HIC selective inhibition of Rev nuclear import mediated by 293T, Jurkat or CEM cytosol or importin β, but not by HeLa, U937 or THP-1 cytosol and transportin and strongly suggest that HIC contributes to the spatial control of Rev function in 293T, Jurkat and CEM cells.

## Discussion

This study identifies the cellular protein HIC as a novel interactor and regulator of HIV-1 Rev nuclear import and function. First, using *in vitro *nuclear import assays, we analysed the role of individual nuclear transport machinery components in mediating Rev nuclear import and employed importin β or transportin as the transport receptors in the presence of a competitive amount of recombinant HIC. We demonstrated that HIC selectively inhibited Rev nuclear import mediated by importin β but not by transportin and that the Rev NLS domain was sufficient and necessary for Rev nuclear import inhibition by HIC. Additional controls and complementary experiments demonstrated that HIC selectively and physically targeted the Rev NLS domain and not the import factors themselves. Furthermore, the observed inhibition of Rev nuclear import did not result from a general block of import pathways or the physical obstruction of the NPC since M9 or Rev nuclear import mediated by transportin remained unaffected by HIC. The molecular recognition of NLSs by import receptors in the cytoplasm determines their nuclear import rate [33,34]. Based on the evidence herein, we propose that the HIC I-mfa domain binds to Rev NLS and selectively prevents its recognition by importin β and subsequent nuclear import proteins. This is similar to I-κB, which interacts with and masks the NF-κB NLS domain, also preventing its nuclear import [35,36]. To extend our study to an *in vivo *cellular context, we next demonstrated that co-expression of HIC and Rev in COS7 cells resulted in the cytoplasmic sequestration of Rev with a concomitant reduction in its nuclear accumulation and this was dependent on the HIC I-mfa domain. Then, using leptomycin B, we further indicated that HIC most likely inhibits Rev nuclear import rather than promoting its nuclear export. This is also consistent with our competitive nuclear import assay performed with COS7 cytosol.

It should be noted that we have employed models where HIC was overexpressed or knocked-down *in vivo*, or added in excess amounts when used with *in vitro *nuclear import assays, and that HIC inhibitory effects on Rev function or Rev nuclear import were dose dependent. Collectively, these observations suggest that HIC acts as a cellular competitor with importin β for binding the Rev NLS, and that the balance between HIC and importin β could influence the rate of Rev nuclear import. Interestingly, we and others have recently described that the expression of HIC is tightly regulated at the transcriptional level [37,38]. Furthermore, compared to 293T where endogenous levels of HIC expression are sufficient to down-modulate HIV-1 Rev activity, HIC is highly expressed in PBMCs and more specifically in HIV-1 target cells, including CD4+ T-cells and monocytes [37]. Additionally, it is also possible that post-transcriptional modification(s) could modulate the relative affinity of Rev NLS for HIC and importin β, and mediate Rev release from sites of sequestration in the cytoplasm. In this regard, Rev is a target of the protein kinase CK2, which phosphorylates Rev at Ser5, Ser8 and results in the conformational change of a region encompassing the NLS domain [39,40]. In addition, Rev Lys33 is also a target of ubiquitination [41].

In the present study, we also attempted to characterise Rev nuclear import pathways within different cellular environments and employed the M9 M peptide, which was specifically designed to inhibit the transportin pathway. Remarkably, HIC and M9 M had opposite effects on Rev nuclear import in a cell type-specific fashion. As previously described by Hutten *et al.*, M9 M inhibited Rev nuclear import in the presence of HeLa cytosolic extracts [23]. Similarly, M9 M but not HIC, inhibited Rev nuclear import using U937 and THP-1 cellular extracts. These results further substantiate the view that transportin and not importin β acts as the major import receptor for Rev in HeLa, U937 and THP-1 cells. Nevertheless, when 293T, Jurkat or CEM cytosolic extracts were employed, HIC but not M9 M inhibited Rev nuclear import, revealing that importin β but not transportin is the dominant nuclear import pathway in these cells. Finally, using a CAT reporter gene assay, overexpression of HIC was shown to reduce Rev activity in 293T, Jurkat and CEM cells. These results were substantiated by siRNA knockdown of endogenous HIC, which remarkably increased Rev activities in 293T cells. In contrast, HIC had no significant effect on Rev function in HeLa, U937 and THP-1 cells. Collectively, these findings support the hypothesis that Rev nuclear import pathways is determined by the cellular context and that importin β and transportin alternate as major import receptors for Rev in a cell specific and mutually exclusive fashion. Remarkably, we also revealed here that while HIC displaces importin β from Rev NLS, Rev could bind simultaneously to both HIC and transportin, strongly suggesting that the molecular determinants for Rev binding to importin β and transportin are different. The multivalent nature of the Rev NLS for multiple import factors would enable Rev to exploit multiple import pathways and to adapt its nuclear trafficking strategy to different cellular environments.

Given the observed effects of HIC on both Tat and Rev localisation and functions, it would be of interest to correlate their respective use of specific nuclear import pathways and the cell specific HIC endogenous level of expression, with HIV-1 replication and to distinguish the effects of HIC-Tat and HIC-Rev interactions on HIV-1 life cycle by employing Tat- or Rev-independent viruses.

Finally, we also describe how HIC interacts and interferes with SV40T NLS nuclear import, which constitutes the archetype of import mediated by the importin α/β pathway. Interestingly, other reports describe interactions of HIC and I-mfa with basic regions. These include the Axin GSK-3 binding domain, and Cyclin T1 KRM, both of which have a high K/R residue content [26,27]. It would be of interest to investigate if HIC could also modulate their nuclear import.

## Conclusions

We have identified HIC as a novel cellular cofactor for the HIV-1 regulatory protein Rev. We propose that the intermolecular masking of Rev NLS by HIC by which HIC control of Rev nuclear import can contribute to the spatial control of its activity. We also show that Rev nuclear import is cell specific and alternatively mediated by transportin or importin β.

## Methods

### DNA constructs and plasmids

pCAGGS-HA-Rev was created by cloning Rev (aa 1-116) sequence into pCAGGS [42]. pFLAG-HIC, pFLAG-HIC(2-144), pFLAG-HIC(144-246) and pDM128-RRE were described previously [25,43]. pGEX-GST-SV40TNLS-GFP, pGEX-GST-importin β, pQE80-RanQ69L, pGEX-GST-YFP, GST-M9 M and His-tagged transportin and importin β vectors were described previously [32,44-47]. pGEX-GST-YFP-M9: M9 sequence encoding hnRNP A1 (aa268-305) was cloned into pGEX-GST-YFP [48]. pGEX-GST-YFP-Rev (aa1-116), pGEX-GST-YFP-RevΔN1 (aa35-116), pGEX-GST-YFP-RevΔN2 (aa46-116) and pGEX-GST-YFP-RevNLS (aa35-46) were generated by cloning Rev relevant sequences into pGEX-GST-YFP (Figure 7A). pBAD/6×His-HIC was described previously [25].

### Western-Blotting analysis

Western-Blotting analysis was performed using BioTrace™PVDF (Pall Corporation) and the SNAP i.d. Protein Detection System (Millipore) according to the manufacturer's instructions. The following primary antibodies were employed: ANTI-MDFIC AB2 and ANTI-FLAG M2 (Sigma); anti_HA High Affinity 3F10 (Roche); Transportin 1 (D45), NTF97/Importin beta (3E9) and Tubulin antibodies (Abcam). The following secondary antibodies (GE Healthcare) were employed: ECL™Anti-mouse IgG and ECL™Anti-rabbit IgG.

### Cell culture and Transfection

293T, HeLa and COS7 cell lines were maintained in Dulbecco's modified Eagle's medium (DMEM) with 0.3 gm/L of L-Glutamine (GIBCO) supplemented with 10% fetal calf serum and antibiotics. Jurkat, CEM, THP-1 and U937 cell lines were cultured in RPMI 1640 medium containing 10% fetal calf serum and supplemented with 0.3mg/L of L-Glutamine (GIBCO) and antibiotics. Transient DNA transfections were performed using FuGENE6 (Roche Diagnostics, Mannheim, Germany) according to the manufacturer's protocol. The total amount of DNA was equilibrated by addition of parent plasmid. Approximately 30,000 HeLa cells and 60,000 293T cells were reverse-transfected with siRNA (30 pmol) in individual wells of a 24 well plate using Lipofectamine™ RNAiMAX (Invitrogen) according to manufacturer's instructions. siRNAs were obtained from QIAGEN: MDFIC_3 (5'-GGAUUGUAGGAGUGGAAGATT-3'), MDFIC_5 (5'GGAGUGAGCUGGCUGGAAATT-3'), MDFIC_7 (5'-CAUGAGAUUUAGCAGACUATT-3') and luciferase GL2 siRNA (negative control). Quantitative real time RT-PCR analysis of HIC mRNA expression was performed 72 hours post-transfection as described before (Gu et al., 2009).

### Nucleofection

Jurkat, CEM, U937 and THP-1 cells were transfected by nucleofection with the Nucleofector device I from Amaxa Biosystems and using the Nucleofector Kit V or Nucleofector Kit C, according the manufacturor's instructions. Total amount of DNA was equilibrated by addition of parent plasmid.

### Purification of Recombinant Proteins

Recombinant GST fusion proteins (GST-YFP Rev/RevΔN1/RevΔN2/RevNLS/M9, GST-SV40TNLS-GFP, GST- importin β, GST-M9M) and His-tagged proteins (RanQ69L, importin β, transportin, HIC) were produced as described previously [25,32,44-47].

### Co-immunoprecipitation Studies

293T cells were transfected with 4 μg of pCAGGS-HA-Rev and/or 1 μg of pFLAG-HIC. 48 hours post-transfection, cells were lysed in 200 μl Native lysis buffer (TBS with 5 mM EDTA PH 8.0, 10 mM NaF, 1% Triton X-100, 10 mM DTT, protein inhibitors). Lysates were incubated with 20 μl of HA Matrix affinity (Roche) at 4°C, overnight and bound complexes were eluted in Leammli sample buffer. Western-Blotting was carried out using Anti-HA High Affinity 3F10 (Roche) or Anti-FLAG M2 monoclonal antibody (Sigma).

### Co-localisation Studies

COS7 cells were transfected with 0.5 μg of pCAGGS-HA Rev, and/or 0.5 μg of pFLAG HIC, pFLAG-HIC(2-144), and/or pFLAG-HIC(144-246). 48 hours post-transfection, cells were fixed in 4% paraformaldehyde and permeabilised with 0.2% Triton X-100. Indirect immnunofluorescence was performed using Anti-HA High Affinity 3F10 (Roche), Anti-FLAG M2 monoclonal antibody (Sigma) as primary antibodies, and anti-rat Ig-Biotin (DAKO), anti-rabbit Ig (Molecular Probes), Streptavidin Texas Red 594 (Molecular Probes) and anti Mouse IgG1 FITC (Molecular Probes) as secondary antibodies. Stained cells were visualized using an Olympus BX50 fluorescent microscope and DP70 camera.

### In vitro nuclear import assay

293T, HeLa, COS7, THP-1, U937, CEM and Jurkat cytosolic extractions were performed as described previously [49]. *In vitro *nuclear import assays and control experiments were performed with HeLa cells as described previously [44]. Competitive *In vitro *nuclear import assays were performed by using GST-YFP-Rev/Rev NLS/RevΔN1/RevΔN2, GST-YFP-M9, GST-SV40TNLS-GFP as nuclear import substrate. RRL, 293T, HeLa, COS7, U937, Jurkat, CEM and THP-1 cytosolic extracts, recombinant N-His importin β or transportin C-His were employed as source of nuclear import factors. The addition of competitive and increasing amount of recombinant 6ΧHis HIC (0.5 to 4 μg) or GST-M9 M (4 μg), were employed for the competition assay. Images were captured using using an Olympus BX50 fluorescent microscope and DP70 camera. Cytosolic extracts (10 μg) were also subjected to Western Blot analysis to monitor the endogenous expression levels of importin β and transportin.

### In vitro "pull down" assay

GST pull down assays were performed as previously described [25]. Competitive *in vitro *GST pull down experiments were performed by adding 4 μg of recombinant N-His importin β or transportin C-His and increasing amount (0.5 to 4 μg) of recombinant 6ΧHis HIC to 0.5 μg of GST-YFP-Rev/Rev NLS immobilized on glutathione sepharose (Amersham).

### Chloramphenicol acetyltransferase (CAT)-ELISA assay

293T and HeLa cells were transfected with 0.1 μg of CAT reporter gene DM128-RRE combined with pCAGGS-HA-Rev (0.05 μg), pFLAG-HIC (1-4 μg) or parent plasmid. Jurkat, CEM, U937 and THP-1 cells were transfected with 1 μg of CAT reporter gene DM128-RRE combined with pCAGGS-HA-Rev (0.5 μg), pFLAG-HIC (4 μg) or parent plasmid. For all cell lines, pRL-TK (Promega) was also transfected to control for transfection efficiency. 24 h post-transfection, the CAT-ELISA assays (Roche) were performed according to the manufacturer's protocols. Alternatively, 24 hours following reverse-transfection of HIC siRNAs (30pmoles) or control Luciferase GL2 (30pmoles) siRNA, cells were transfected with DM128-RRE (0.2 μg), pRL-TK (0.02 μg) and pCAGGS-HA-Rev (0.02 μg) or its parent plasmid and CAT assay was performed 48 hours post-transfection. Cell lysates were also subjected to Western Blot analysis to monitor HIC and Rev expression levels.

## Competing interests

The authors declare that they have no competing interests.

## Authors' contributions

LG conducted experimental procedures, data interpretation, and contributed to the experimental design and to drafting the manuscript. TT contributed to reagents and to study design. MAJ and GPY participated in the experimental procedures. NS contributed to the interpretation of results and final editing of the manuscript. WWH co-supervised the study design, execution and analysis and revised the manuscript critically. VG conceived and designed the study, co-supervised its execution and analysis and drafted the manuscript. All the authors read and approved the manuscript.

## Supplementary Material

Additional file 1 **Supplementary Figure 1: HIV-1 Rev dominant nuclear import pathways are cell specific. Nuclear import of GST-YFP-Rev, GST-YFP-M9 and GST-SV40T NLS-GFP were examined using *in vitro *nuclear import assays**. Digitonin permeabilized HeLa cells were incubated with 10 μl of reaction mixtures containing 1 μg of an import substrate, ATP regeneration system, and THP-1/CEM/COS7 cytosolic extracts. Recombinant 6×His-HIC at 0.5, 1 or 2 μg was added. Rev nuclear signal intensities were analyzed by ImageJ for a minimum of 100 cells and illustrated by box plots. Statistical significance analysis was performed with a two-tailed unpaired Student's *t *test *, *P *< 0.05; **, *P *< 0.01; ***, *P *< 0.001 Click here for file

Additional file 2 **Supplementary Figure 2: Endogenous expression levels of importin-β, transportin and HIC in HeLa, 293T, COS7, Jurkat, CEM, THP-1, U937 cytosolic extracts**. The seven different cytosolic extracts (10 μg) employed for the *in vitro *nuclear import assay were tested by WB for Importin-β, transportin and HIC endogenous expression levels. Click here for file

Additional file 3 **Supplementary Figure 3: Rev expression remains unaffected following the modulation of HIC expression**. **A. Rev expression remains constant following HIC over-expression**. Corresponding lysates of 293T or Hela cells transfected with 0.1 μg of pDM128-RRE combined with 0.05 μg of HA-Rev and 4 μg of FLAG-HIC were analysed by Western-Blot (WB) for HIC and Rev expression. α-Tubulin was employed as a loading control. **B. siRNA-mediated knockdown of HIC has no effect on Rev expression**. Corresponding lysates of HeLa and 293T cells, reverse-transfected with three distinct HIC siRNAs or negative control, and subsequently transfected with DM128-RRE, pRL-TK and pCAGGS-HA-Rev or its parent plasmid were analysed by WB to monitor the expression levels of HA-Rev and endogenous HIC. α-Tubulin was employed as a loading control. Click here for file
