# Supplementary figures and images for: Intermolecular masking of the HIV-1 Rev NLS by the cellular protein HIC: Novel insights into the regulation of Rev nuclear import
Source: Retrovirology. 2011 Mar 14;8:17. doi: 10.1186/1742-4690-8-17 (PMC3062594; doi:10.1186/1742-4690-8-17)

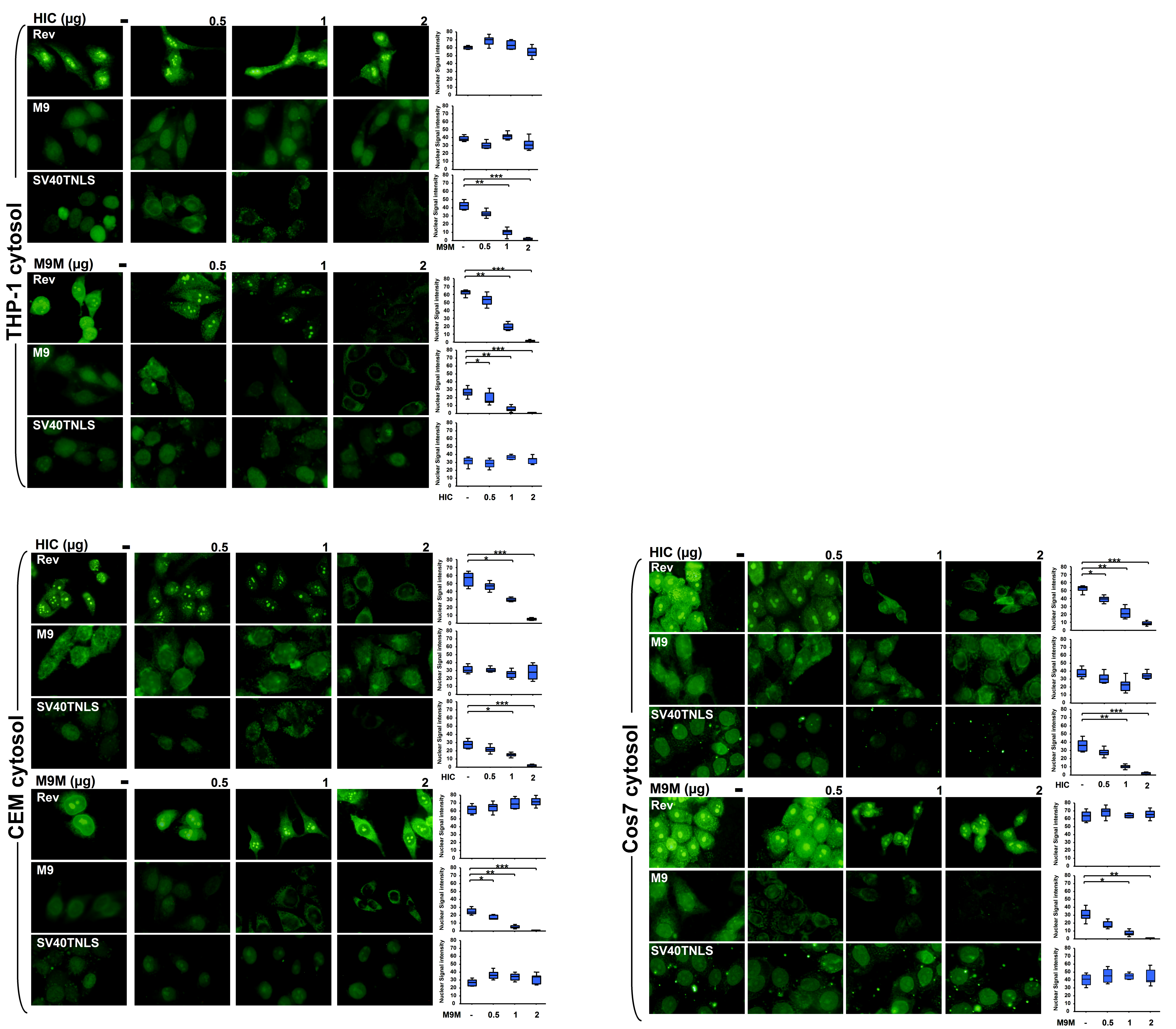

Supplement: Additional file 1 — Supplementary Figure 1: HIV-1 Rev dominant nuclear import pathways are cell specific. Nuclear import of GST-YFP-Rev, GST-YFP-M9 and GST-SV40T NLS-GFP were examined using in vitro nuclear import assays. Digitonin permeabilized HeLa cells were incubated with 10 μl of reaction mixtures containing 1 μg of an import substrate, ATP regeneration system, and THP-1/CEM/COS7 cytosolic extracts. Recombinant 6×His-HIC at 0.5, 1 or 2 μg was added. Rev nuclear signal intensities were analyzed by ImageJ for a minimum of 100 cells and illustrated by box plots. Statistical significance analysis was performed with a two-tailed unpaired Student's t test *, P < 0.05; **, P < 0.01; ***, P < 0.001 [file 1742-4690-8-17-S1.TIFF]

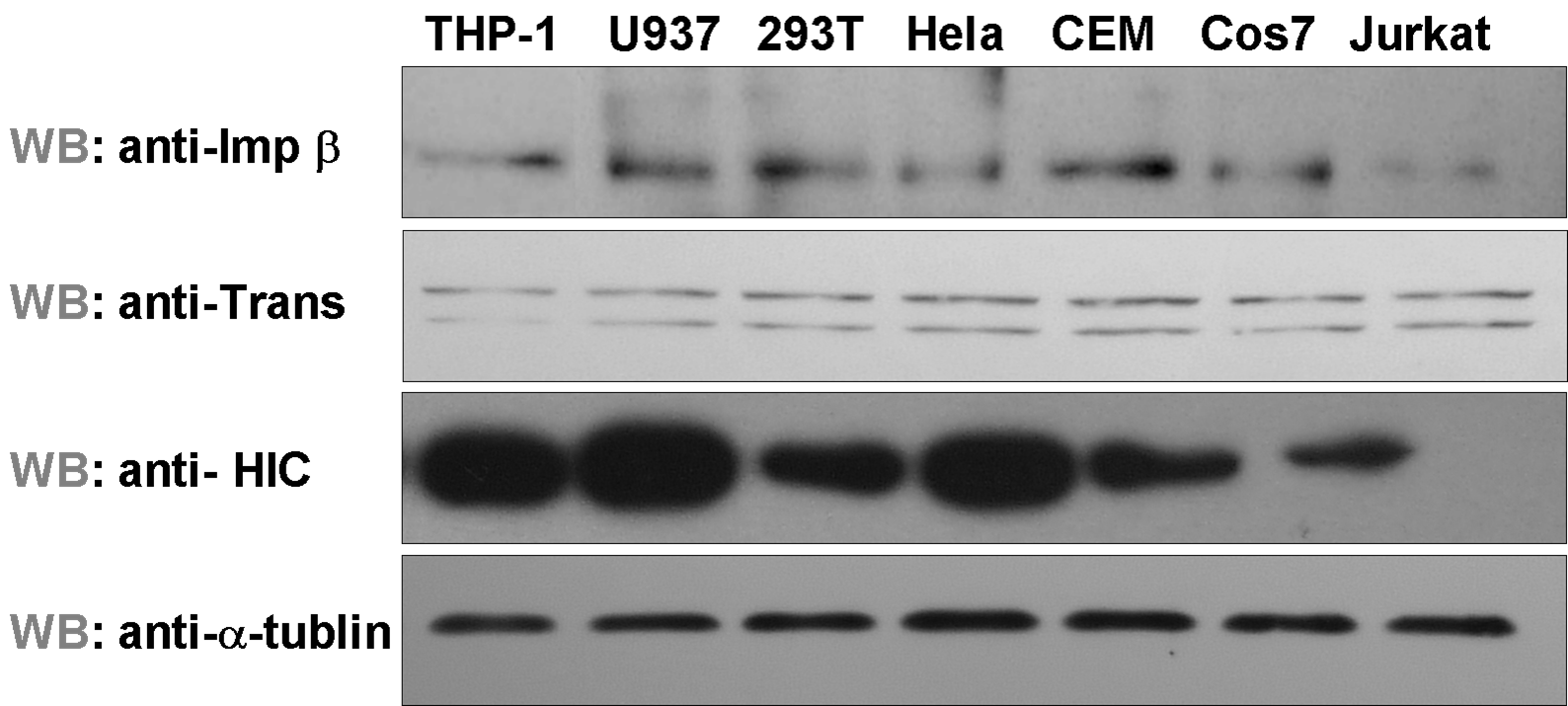

Supplement: Additional file 2 — Supplementary Figure 2: Endogenous expression levels of importin-β, transportin and HIC in HeLa, 293T, COS7, Jurkat, CEM, THP-1, U937 cytosolic extracts. The seven different cytosolic extracts (10 μg) employed for the in vitro nuclear import assay were tested by WB for Importin-β, transportin and HIC endogenous expression levels. [file 1742-4690-8-17-S2.TIFF]

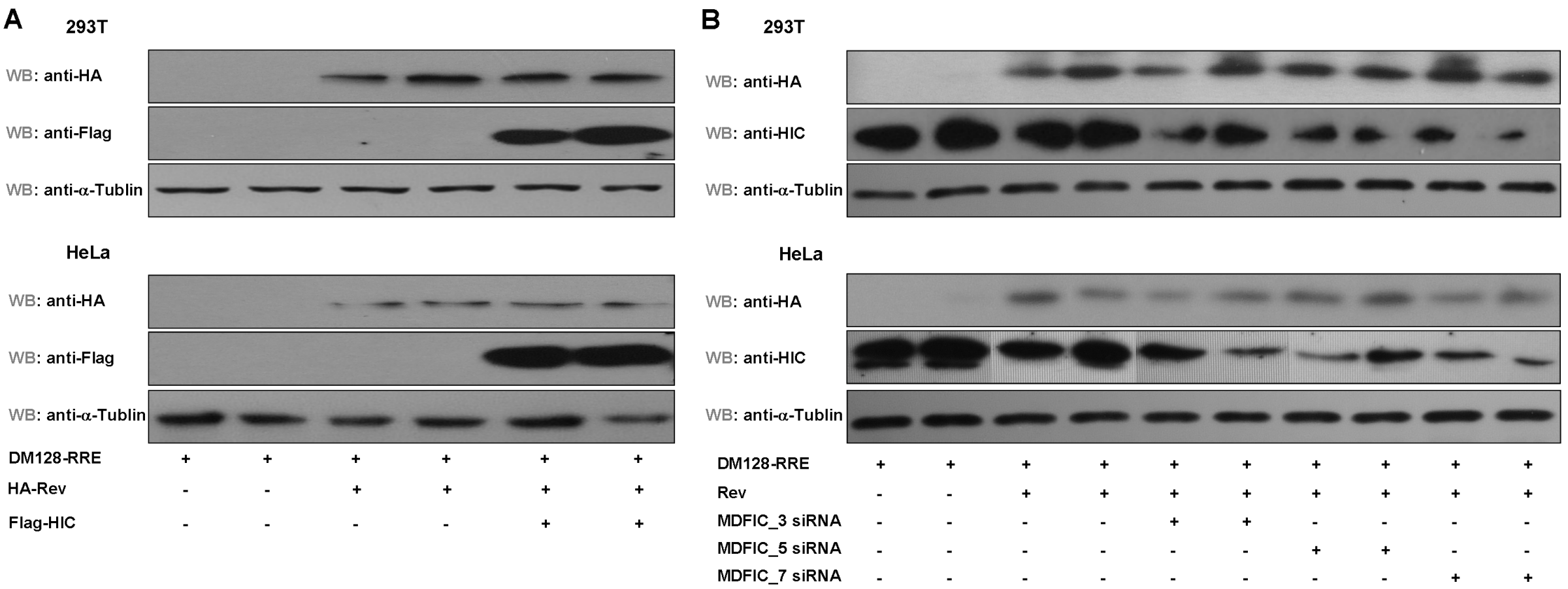

Supplement: Additional file 3 — Supplementary Figure 3: Rev expression remains unaffected following the modulation of HIC expression. A. Rev expression remains constant following HIC over-expression. Corresponding lysates of 293T or Hela cells transfected with 0.1 μg of pDM128-RRE combined with 0.05 μg of HA-Rev and 4 μg of FLAG-HIC were analysed by Western-Blot (WB) for HIC and Rev expression. α-Tubulin was employed as a loading control. B. siRNA-mediated knockdown of HIC has no effect on Rev expression. Corresponding lysates of HeLa and 293T cells, reverse-transfected with three distinct HIC siRNAs or negative control, and subsequently transfected with DM128-RRE, pRL-TK and pCAGGS-HA-Rev or its parent plasmid were analysed by WB to monitor the expression levels of HA-Rev and endogenous HIC. α-Tubulin was employed as a loading control. [file 1742-4690-8-17-S3.TIFF]
